# Microtiter miniature shaken bioreactor system as a scale-down model for process development of production of therapeutic alpha-interferon2b by recombinant *Escherichia coli*

**DOI:** 10.1186/s12866-017-1145-9

**Published:** 2018-01-04

**Authors:** Joo Shun Tan, Sahar Abbasiliasi, Saeid Kadkhodaei, Yew Joon Tam, Teck-Kim Tang, Yee-Ying Lee, Arbakariya B. Ariff

**Affiliations:** 1 Bioprocess Technology, School of Industrial Technology, Universiti Sains Malaysia, 11800 Gelugor, Pulau Pinang Malaysia; 2 Department of Microbiology, Faculty of Biotechnology and Biomolecular Sciences, Universiti Putra Malaysia, 43400 UPM Serdang, Selangor Malaysia; 3 Institute of Tropical Agriculture, Universiti Putra Malaysia, 43400 UPM Serdang, Selangor Malaysia; 4 Department of Bioprocess Technology, Faculty of Biotechnology and Biomolecular Sciences, Universiti Putra Malaysia, 43400 UPM Serdang, Selangor Malaysia; 5 Institute of Bioscience, Universiti Putra Malaysia, 43400 UPM Serdang, Selangor Malaysia

**Keywords:** Microscale, Automated system, α-interferon2b, Fermentation, Extraction

## Abstract

**Background:**

Demand for high-throughput bioprocessing has dramatically increased especially in the biopharmaceutical industry because the technologies are of vital importance to process optimization and media development. This can be efficiently boosted by using microtiter plate (MTP) cultivation setup embedded into an automated liquid-handling system. The objective of this study was to establish an automated microscale method for upstream and downstream bioprocessing of α-IFN2b production by recombinant *Escherichia coli*. The extraction performance of α-IFN2b by osmotic shock using two different systems, automated microscale platform and manual extraction in MTP was compared.

**Results:**

The amount of α-IFN2b extracted using automated microscale platform (49.2 μg/L) was comparable to manual osmotic shock method (48.8 μg/L), but the standard deviation was 2 times lower as compared to manual osmotic shock method. Fermentation parameters in MTP involving inoculum size, agitation speed, working volume and induction profiling revealed that the fermentation conditions for the highest production of α-IFN2b (85.5 μg/L) was attained at inoculum size of 8%, working volume of 40% and agitation speed of 1000 rpm with induction at 4 h after the inoculation.

**Conclusion:**

Although the findings at MTP scale did not show perfect scalable results as compared to shake flask culture, but microscale technique development would serve as a convenient and low-cost solution in process optimization for recombinant protein.

## Background

Recent advances in life sciences and the establishment of high throughput screening technologies have led to the identification of large numbers of potentially important biopharmaceuticals. Many of these products are now in the later stages of product development and a priority is put forth to overcome the challenges faced in the difficulty of identifying large number of new drug candidates without contributing to a significant increase in bioprocess development costs. Conventionally, shake flask cultures were used at laboratory scale and in the bio-industry for screening and bioprocess development purposes. However, the screening using shake flask culture is still considered as labor-intensive and insufficient to evaluate a large number of samples especially during bioprocess development [1]. As a consequence, application of microscale bioreactors with volumes ranging from microliter to milliliter for the cultivation of microorganisms is gaining acceptance in both academic research and industrial applications. The microscale fermentation system, such as 96-deep well plates, can be used for the screening of medium compositions and clones as well as for up-scaling, optimization and validation of the process [2].

The microscale fermentation technique, especially with automated system integration, is considered as a more rapid system that can be used to accelerate drug discovery by means of high-throughput screening of multiple drug targets [3]. The ability of the automated microscale system to perform representative process studies at small scale level with minimal human intervention would indeed help in reducing production and development costs.

Alpha-interferon-2b (α-IFN2b), which is known to aid in curing diseases such as chronic hepatitis C and chronic hepatitis B [4] was used as the model to investigate the feasibility of its production from *Escherichia coli* using microscale fermentation [5]. In order to assess the reliability of miniature high throughput systems as a tool for scale-down studies, it is critical to demonstrate their ability to mimic the conditions and productivity that would be expected at larger scales. A majority of the miniature systems that have been reported over the years have not done this [6]. The miniature bioreactor systems described herein are demonstrated in replacing the traditionally popular shake flask and manual osmotic shock treatment for parallel, high throughput operation, by optimizing the fermentation parameters with α-IFN2b production as a model. So far, report on the use of automated osmotic shock treatment is not available in the literature.

The main objective of this study was to find a low-cost solution in process optimization of α-IFN2b production by recombinant *E. coli* Rosetta-gami2 (DE3) using microtitre plates that provide a miniaturised system and high throughput solution that is amenable to automation. In this respect, four fermentation parameters in MTP viz. inoculum size, agitation speed and working volume and induction profiling were studied. The growth of recombinant *E. coli* Rosetta-gami2 (DE3) in MTP and shake flask was modelled to evaluate the high-throughput fermentation by replacing shake flask in screening and optimization studies using MTP integrated with automated liquid handling robotic system. This is the first report on the extraction of α-IFN2b by osmotic shock using automated microscale platform.

## Methods

### Microorganism and inoculum preparation

Recombinant *E. coli* strain Rosetta-gami2 (DE3), previously constructed to express α-IFN2b in the periplasm, was used in this study [7]. The pET26b-IFN contained the *pel*B (pectate lyase B) signal sequence for the transfer of ligated gene product (α-IFN2b) in the periplasm of *E. coli*. Stock cultures were kept at -80 °C in 10% (*v*/v) glycerol and the inoculum was prepared by inoculating 2% (v/v) of stock culture into a 250 mL baffled shake flask containing 50 mL of Terrific Broth (TB) supplemented with 30 mg/L kanamycin sulphate (Sigma Aldrich,. St Louis, MO, USA) and 34 mg/L chloramphenicol (Sigma Aldrich,. St Louis, MO, USA). The inoculated flasks were then incubated at 37 °C in Certomat incubator shaker (B. Braun Medical, Melsungen, Germany), agitated at 250 rpm for 16 h. This culture was used as a standard inoculum for all fermentations. TB was used in all fermentations for the production of α-IFN2b with an inoculum size of 8% (*v*/v). Prior to usage, the broth was autoclaved at 121 °C, 15 psi for 15 min.

### Automated microscale platform

MTP cultivations were carried out in 96-deep well plates (volume size = 1.2 mL) (Deep Well Plate, Masterblock, round bottom, polypropylene obtained from Greiner Bio-One, Germany) covered with a sterile gas permeable adhesive sealing film with pore size of 0.2 μm (AirPore™ tape sheet, Qiagen, Mississauga ON, Canada) to allow oxygenation of the cell culture and integrated with automated liquid handling robotic system (Tecan Freedom EVO, Tecan Schweiz AG, Switzerland). Culture medium and inoculum were added into the wells of MTP using automated eight channel liquid handler which was controlled by Freedom EVO system. The automated liquid handling robotic system mainly consisted of a vacuum separation unit (Te-VacS, Tecan Schweiz AG, Switzerland), an eight channels liquid handler (LIHA, Tecan Schweiz AG, Switzerland), a microplate carrier (ROMA, Tecan Schweiz AG, Switzerland) and an orbital mixer (Te-Shake, Tecan Schweiz AG, Switzerland). The automated microscale extraction and filtration techniques were developed by the use of a Tec-VacS vacuum filtration manifold which was designed for rapid separation of biological substances.

### Experimental procedures

#### Fermentation using automated microscale platform

The effectiveness of MTP cultivation for the optimization of fermentation process was performed by assessing several important parameters. The effects of working volume ranged from 50% (*v*/v) to 80% (v/v) and shaking speed ranged from 400 rpm to 1000 rpm were first evaluated. Subsequently, the effects of other cultivation parameters including inoculum size (2% v/v-8% v/v) and induction time (4 h–10 h) were evaluated using the optimal working volume and agitation speed. All fermentations in MTP were inoculated with 8% (v/v) inoculum and incubated at 37 °C for 4 h unless stated otherwise. In order to evaluate the possibility of replacing shake flask system for its use in screening and optimization studies with the automated microscale platform, growth profiles and α-IFN2b production patterns obtained from the platform was further validated by performing the cultivations in 250 mL shake flask (Thermo Fisher Scientific, Inc., MA, USA). The shake flask cultures were incubated in an incubator shaker at 37 °C and agitated at 250 rpm for 4 h. After the active growth was achieved, the cultures were further induced with 1 mM of isopropyl β-D-1-thiogalactopyranoside (IPTG) and the cultivation temperature was immediately switched to 30 °C. The samples were then harvested at predetermined time intervals.

Osmotic shock extraction using automated microscale platform.

After the completion of the fermentation in MTP, the cells were harvested by removing the culture supernatant, followed by extraction of α-IFN2b using protein extraction buffer with the aid of an automated liquid handling robotic system according to a modified osmotic-shock method adapted from Tan et al. [7]. In this procedure, the culture from MTP was transferred by the liquid handling system to a 1 mL microwell filter plate (AcroPrep™ 96-well Filter Plates, Pall Corp., USA), which was run automatically on the Te-VacS unit consisted of a three ways valve. This valve was used to vent the plate manifold at the maximum negative pressure difference (700 mbar). As a result of the pressure difference, the culture supernatant was filtered through the microwell filter plate to the collection plate or beneath the filter plate. This step enables the measurement of the filtrate and permeate mass. Thereafter, the osmotic shock extraction buffer (20% (*w*/*v*) sucrose, 33 mM Tris, and 5 mM EDTA at pH 8) was dispensed into the filter plate containing the harvested recombinant *E. coli* Rosetta-gami2 (DE3) at zero pressure difference on the Te-VacS unit. The mixture was then further incubated at 30 °C with shaking for 5 min to enhance the osmotic shock process. After incubation, the buffer was then discarded using Te-VacS unit and the shrunk cells generated were rapidly re-suspended in ice-cold distilled water to extract α-IFN2b. Subsequently, the extracted α-IFN2b in distilled water was then filtered out and collected at the collection plate for analysis.

### Manual osmotic shock extraction

In manual osmotic shock extraction, the culture sample was first centrifuged at 8000 x g for 10 min. The cell pellet was re-suspended in osmotic shock extraction buffer. The mixture was then incubated at 30 °C with shaking for 5 min to enhance osmotic shock. Subsequently, the mixture was centrifuged at 8000 x g for 10 min and the supernatant was discarded. The shrunk cells were re-suspended in ice-cold distilled water and incubated with shaking for 15 min. The mixture was then centrifuged at 8000 x g for 10 min and the supernatant was collected for analysis.

### Kinetic models and parameters assessment

The kinetic elements of the processes were evaluated to compare the production of IFN-α2b by recombinant *E. coli* Rosetta-gami2 (DE3) in shake flask and MTP cultures. Unstructured models, based on Logistic equation, was used for the modelling of growth rate of *E. coli* using Sigmaplot 11.0. The generic logistic eq. [8] can be customarily written as eq. 1:

1 $$ \boldsymbol{X}=\frac{{\boldsymbol{X}}_{\mathbf{0}}\boldsymbol{\exp}\left(\boldsymbol{\mu} \boldsymbol{t}\right)}{\Big(\mathbf{1}-\left(\frac{{\boldsymbol{X}}_{\mathbf{0}}}{{\boldsymbol{X}}_{\boldsymbol{m}}}\right)\left(\mathbf{1}-\boldsymbol{\exp}\left(\boldsymbol{\mu} \boldsymbol{t}\right)\right)} $$

X_0_ and X_max_ is the initial and maximum cell concentration (g/L) and μ refers to the maximum specific growth rate (h^−1^).

Yield and volumetric productivity are given by

2 $$ Specific yield=\frac{Maximum\alpha IFN2b\ \left(\mu g/L\right)}{\mathrm{Dry}\ \mathrm{cell}\  \mathrm{weight}\ \left(\mathrm{g}/\mathrm{L}\right)} $$

3 $$ \boldsymbol{Volumetric}\boldsymbol{productivity}=\frac{\boldsymbol{Maximum}\boldsymbol{\alpha } \boldsymbol{IFN}\mathbf{2}\boldsymbol{b}\ \left(\boldsymbol{\mu} \boldsymbol{g}/\boldsymbol{L}\right)}{\boldsymbol{fermentationtime}\ \left(\boldsymbol{h}\right)} $$

### Analytical methods

Dry cell weight (DCW) was determined by filtration and oven-dry method [7]. Cell concentration was also quantified by optical density (OD). The relationship between dry cell weight and OD_600_ was observed as 0.278 g DCW/L/OD_600_. The quantification of α-IFN2b was performed using fully automated surface plasmon resonance detection system (BIAcore 3000, GE HealthCare) [7].

## Results

### Comparison of manual and automated microscale platform osmotic shock extraction systems

Osmotic shock extraction of α-IFN2b was performed manually and also with the use of the automated microscale platform. The results of α-IFN2b release tests, which compare the consistency of the two different extraction methods are shown in Table 1. The highest quantity of α-IFN2b released by osmotic shock extraction using the automated microscale platform (49.2 μg/L) was comparable to that of α-IFN2b released by the manual extraction (48.8 μg/L). However, the standard deviation for the result of the automated microscale platform was 2 times lower than the result of the conventional method.

**Table 1:** Comparison of two different methods of osmotic extraction of α-IFN2b, manual and automated microscale platform

Type of osmotic shock extraction	DCW	α-IFN2b release	Volume of sample
	(g/L)	(μg/L)	(mL)
Automated	3.05 ± 0.08	49.2 ± 0.55	1
Manual	2.94 ± 0.12	48.8 ± 1.34	1

The results of DCW and α-IFN2b release are the average of triplicate experiments

### Influence of inoculum size in MTP fermentations

The effect of inoculum size on α-IFN2b production by recombinant *E. coli* Rosetta-gami2 (DE3) is shown in Table 2. At a constant agitation speed (800 rpm), working volume (80%) and induction time (4 h), growth of *E. coli* was increased from 2.83 g/L to 3.1 g/L with increasing inoculum size from 2% to 8% (*v*/v). The highest volumetric productivity (4.95 μg/L/h) and yield (15.9 μg/g cell) were obtained at an inoculum size of 8% (v/v). The addition of 8% (v/v) inoculum size also yielded the highest production of α-IFN2b (49.5 μg/L) in the MTP automated cultivation system, which was two times higher than that obtained in the cultivation with inoculum size of 2% (v/v) (25.7 μg/L). Results from this study showed that the inoculum size has greater influence to α-IFN2b production than growth of *E. coli.*

**Table 2:** Effect of inoculum size on α-IFN2b production by *E. coli* in MTP fermentations

Inoculum size	Maximum DCW	Time to achieve maximum DCW	Maximum α-IFN2b	Fermentation time	Specific yield	Volumetric productivity
% (v/v)	(g/L)	(h)	(μg/L)	(h)	(μg/g cell)	(μg/L/h)
MTP						
2	2.83 (0.01)	8	25.7 (0.01)	10	9.1	2.57
4	2.89 (0.03)	8	31.4 (0.03)	10	10.9	3.14
6	2.95 (0.03)	8	37.3 (0.01)	10	12.7	3.73
8	3.10 (0.01)	8	49.5 (0.01)	10	15.9	4.95

The results of maximum DCW and maximum α-IFN2b are the average of triplicate experiments. The value in bracket is the standard deviation. Specific yield and volumetric productivity are calculated with the average values. Fermentation time is the time taken from inoculation to reach a maximum concentration of α-IFN2b

### Profiling of optimal induction conditions in MTP fermentations

IPTG was added at different times during the active growth phase to evaluate the effect of induction time on the expression of α-IFN2b (Table 3). The highest production α-IFN2b in MTP (50.4 μg/L) was obtained when the culture was induced at 4 h and a decline in α-IFN2b production was observed when the induction time was performed at 6 h, 8 h and 10 h. The highest yield (16.5 μg/g cell) and volumetric productivity (5.04 μg/L/h) were also achieved at the induction time of 4 h.

**Table 3:** Effect of induction points on α-IFN2b production by *E. coli* in MTP fermentations

Induction point	Maximum DCW	Time to achieve maximum DCW	Maximum α-IFN2b	Fermentation time	Specific yield	Volumetric productivity
(h)	(g/L)	(h)	(μg/L)	(h)	(μg/g cell)	(μg/L/h)
MTP						
4	3.05 (0.08)	8	50.4 (0.31)	10	16.5	5.04
6	3.64 (0.04)	8	29.2 (0.01)	12	8.0	2.43
8	4.37 (0.06)	8	32.2 (0.04)	14	7.4	2.30
10	5.02 (0.06)	8	34.8 (0.04)	16	6.9	2.18

The results of maximum DCW and maximum α-IFN2b are the average of triplicate experiments. The value in bracket is the standard deviation. Specific yield and volumetric productivity are calculated with the average values. Fermentation time is the time taken from inoculation to reach a maximum concentration of α-IFN2b

### Influence of agitation speed in MTP fermentations

**Fig. 1:**
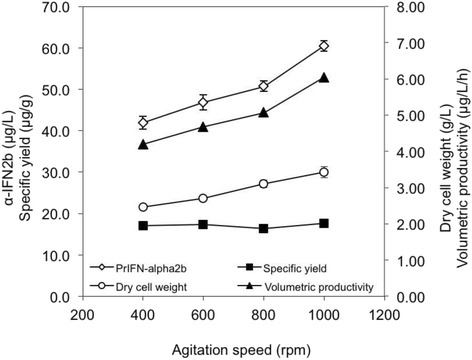
Effects of different agitation speeds on growth of *E. coli* Rosetta-gami2 (DE3) and the ability to produce α-IFN2b in MTP fermentation

The influence of agitation speed on growth of recombinant *E. coli* Rosetta-gami2 (DE3) and α-IFN2b production in MTP is shown in Fig. 1. The highest growth of *E. coli* (3.43 g/L) and production of α-IFN2b (60.5 μg/L) were observed at agitation speed of 1000 rpm, for MTP fermentation where inoculum size, working volume and induction time were fixed at 8% (*v*/v), 80% and 4 h, respectively. Reduced production of α-IFN2b (41.9 μg/L) was observed in MTP fermentation at 400 rpm.

### Influence of working volume in MTP fermentations

**Fig. 2:**
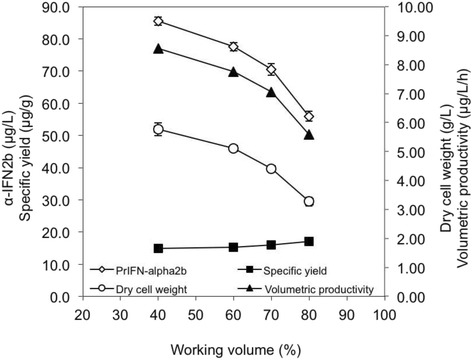
Effects of different working volumes on growth of *E. coli* Rosetta-gami2 (DE3) and the ability to produce α-IFN2b in MTP fermentation

The influence of working volume on growth of recombinant *E. coli* Rosetta-gami2 (DE3) and α-IFN2b production in MTP is shown in Fig. 2. Reduced growth of *E. coli* and production of α-IFN2b were observed with increasing working volume from 40% to 80%. The production of α-IFN2b in MTP with a working volume of 40% (85.5 μg/L) was substantially higher than that obtained in MTP with a working volume of 80% (55.9 μg/L).

### Growth profiles and α-IFN2b production patterns in MTP and shake flask

**Fig. 3:**
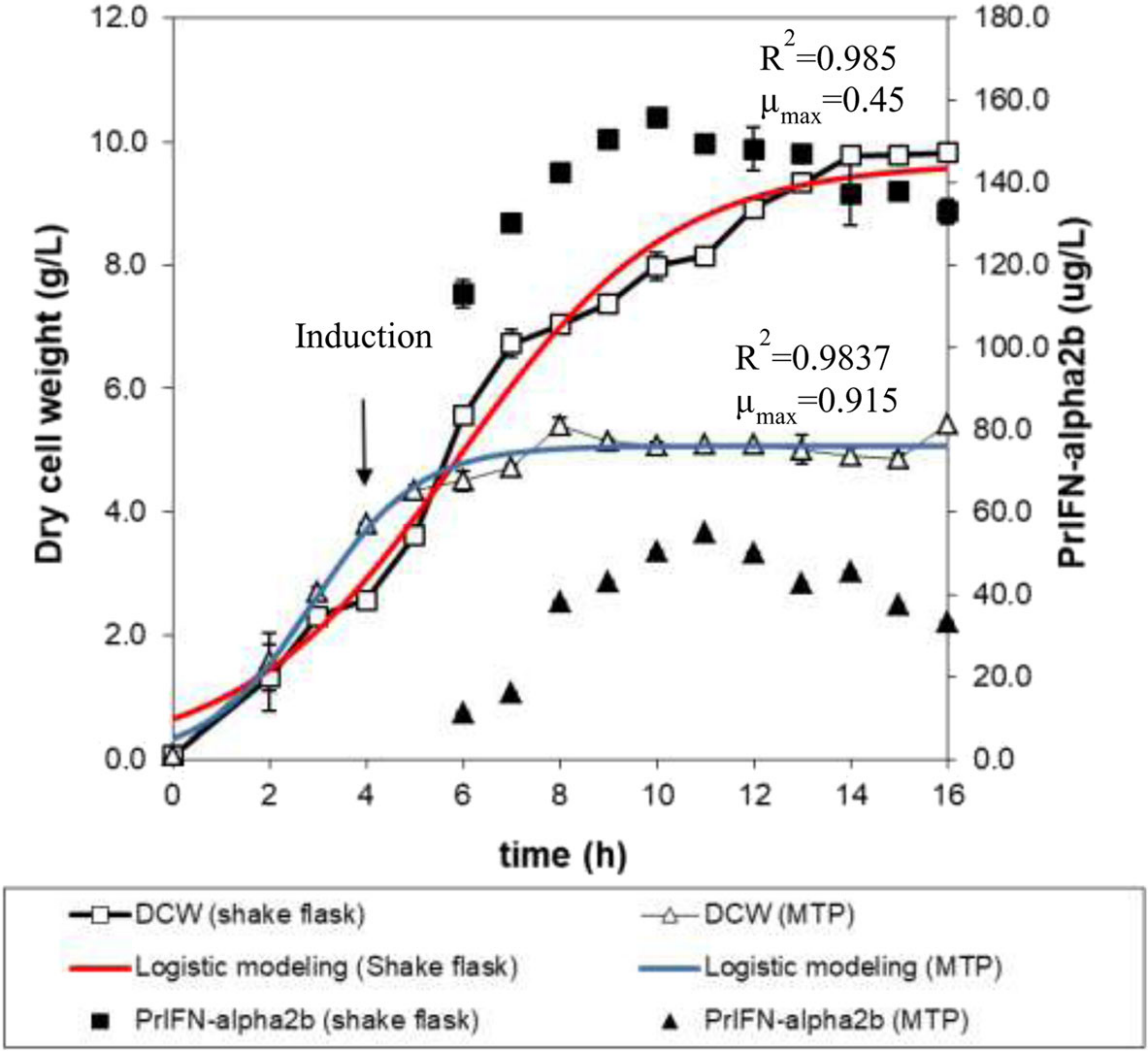
Time course of *E. coli* Rosetta-gami2 (DE3) fermentation for α-IFN2b production in MTP and shake flask systems

The optimal fermentation conditions in MTP were subsequently compared with shake flask fermentation for α-IFN2b production (Fig. 3). In both fermentation systems tested in this study, the concentration of *E. coli* cells and α-IFN2b production was concomitantly increased with fermentation time. Logistic equation was found sufficient to describe growth of *E. coli* in both fermentation systems, where the calculated data fitted well to the experimental data. The specific growth rate was decreased with fermentation time and reached to very low specific growth rate (<0.05 h^−1^) after 6 h and 9 h for fermentation in both MTP and shake flask. The maximum or initial specific growth rate of *E. coli* cultivated in MTP and shake flask was 0.915 h^−1^ and 0.45 h^−1^, respectively.

## Discussion

Current methods of osmotic shock extraction, which requires many steps did not allow for large capacity automated processing. The procedure is cumbersome due to the involvement of a number of centrifugation steps. Therefore, it is difficult to use this procedure to screen large numbers of samples rapidly. Periplasmic protein extraction is becoming a bottleneck in limiting the number and types of culture samples to be analysed. The application of automated high-throughput microscale osmotic shock extraction techniques has the potential to address and expand this capability. Hence, an automated high-throughput system was developed in this study that prepared osmotic shock extracted α-IFN2b from periplasmic region of *E. coli* cell for large capacity processing. Cell harvesting and osmotic shock extraction were performed in 96-well microtiter plate from which extracted α-IFN2b samples were collected. The automated microscale platform provided an opportunity for minimal human interaction, which in turn, reduce the chances for human errors to occur due to the use of automation together with the concurrent runs of the multiple samples in the same environment.

For cultivation with small inoculum sizes, the cultures need to adapt its cellular respiration before entering the active growth stage [9], suggesting that the bacterial cultures will experience a long lag period. Results from this study have demonstrated that the inoculum size plays an important role in the optimization of recombinant α-IFN2b production. The effect of inoculum size was suggested to be related to the length of the lag phase in the cultivation, which resulted in different cell densities of bacterial expression [10]. Small inoculum size may cause slow adaptation of *E. coli* culture when inoculated into a new fermentation medium [11] resulted in a long lag phase with insufficient number of cells that would reduce the production of α-IFN2b.

The induction time is the switch point between cell growth and recombinant protein synthesis by *E. coli*. The transcription of the target gene will begin upon IPTG induction [12]. Commonly, the optimum point for induction was at mid-log phase. In some instance, induction was also performed at a stationary phase [13]. Sandén et al. [14] investigated the limiting factors in *E. coli* when induced at different induction times with respect to specific growth rate. They claimed that the ribosome was degraded upon induction at high specific growth rates, which occurred at active growth phase due to high production level of protein. Induction at a lower specific growth rates (late-log phase and stationary phase) would make carbon (energy) to be a limiting factor. Similar findings were also obtained in this study. High energy is required for the translocation process of proteins into periplasm. Therefore, the induction at the early active growth phase (4 h) was preferred for improvement of α-IFN2b production. This is in agreement with our previous report [15], where the highest α-IFN2b production in shake flask was obtained when the culture was induced at 4 h. In *E. coli* fermentation for α-IFN2b production, as reported in our previous study, the amount of acetate accumulated in the culture was increased with fermentation time. During the early stages of fermentation, acetyl CoA pathway was used heavily by *E. coli* to enhance growth rate, which resulted in higher consumption of glucose and induction of the acetate secretion [15]. During the later stages of fermentation, growth of *E. coli* was inhibited by high concentration of acetate accumulated in the culture, resulted in the reduction of α-IFN2b production [16]. Therefore, induction at the early stages of fermentation was preferred to avoid high accumulation of acetate in the culture at the later stages of fermentation.

Suitable agitation leads to sufficient supply of dissolved oxygen in the culture. Cultivation in MTP often fails to reach high cell density under normal agitation, presumably due to limitations in oxygen transfer rate. Thus, in order to provide sufficient oxygen transfer from gas to liquid phase in a 96-deep well plate, a more vigorous shaking condition is expected for attaining high growth rates and α-IFN2b production [17–19]. The findings from this study is consistent with the results reported by Lee [20]**,** who claimed that oxygen starvation could inhibit growth rate of *E. coli* and reduction in rate of product formation.

Oxygen-transfer capacity in MTP relies mainly upon the specific surface area for oxygen transfer [21]. Ramirez-Vargas et al. (2014) claimed that the oxygen transfer rate was inversely proportional to fill volume, particularly at higher shaking frequencies in microtiter plates, which affected the cell growth and protein production. Although larger volume of culture initially contains more oxygen, nutrients and space for growth of bacterium, the void in the container and subsequent oxygenation of the culture would eventually deplete the oxygen transfer capability in the culture. On the contrary, high air exchange rate in the headspace of MTP enhanced α-IFN2b production, where a large headspace provided better oxygenation for the cells grown in MTP wells [22].

Micheletti et al. [23] compared the microbial and mammalian fermentations as well as biotransformation in MTPs with those in a stirred tank bioreactor. Their research, however, was based on laborious and error-prone sampling methods from the different reactor scales. Therefore, it could be suggested that the automated MTP would be an ideal platform for all tasks in fermentation science and bioprocess development if the automation of sampling and processing in upstream and downstream are possible and easier to perform. From the fermentation time course, it was observed that the automated MTP could be used to replace shake flask cultures in screening and optimization of the process. Hevekerl et al. [24] have investigated a scale-down of itaconic acid production by *Aspergillus terreus* from shake flask to microtiter plate. They found that the itaconic acid production in microtiter plates was comparable to that obtained in shake flask culture. Knorr et al. [25] carried out automated set up with 48 parallel stirred bioreactors at a milliliter scale (10 mL) for the production of riboflavin by *Bacillus subtillis*. However, the automated system set up by them, required more medium volume and did not link to the downstream processing. Jackson et al. [26] established a microscale normal flow filtration (NFF) technique to harvest *E. coli* TOP10 cells using a custom designed 8–24-well filter plates and a commercial 96-well multiscreen filter plates. Optimization of upstream and downstream operations was clearly integrated in their technique. In this study, the automated microscale filtration was extended to microscale osmotic shock treatment, so that the crude recombinant protein (α-IFN2b) could be obtained at the completion of the process.

## Conclusion

Results from the present study provided ample evidences to claim that automated high-throughput microscale osmotic shock extraction techniques that integrated to automated MTP system was capable to prepare crude α-IFN2b from periplasmic region of recombinant *E. coli* cells. This system could be used for large capacity processing with high reproducibility with the standard deviation of 2 times lower than the manual method. The automated microscale MTP system could also provide a high-throughput method for optimizing the production of α-IFN2b by recombinant *E. coli*. The automated MTP system has capability in optimizing cultivation parameters such as inoculum size, induction time, working volume and agitation speed with enhanced growth of *E. coli* (5.4 g/L) and the production of α-IFN2b (85.5 μg/L). Substantial differences in the maximum specific growth rates of *E. coli* cultivated in two different systems in this study, as calculated using Logistic models suggests that the automated microtiter-based system could not perfectly replace with shake flask. However, microscale technique development would serve as a convenient and low-cost solution in process optimization for recombinant protein.

## Abbreviations

DCW: Dry cell weight
MTP: Microtiter plate
OD: Optical density
*pel*B: Pectate lyase B
TB: Terrific broth
α-IFN2b: alpha-interferon-2b
